# Clinical Pharmacy Faculty Provision of Direct Patient Care, Challenges, and Opportunities

**DOI:** 10.3389/fmed.2023.1143576

**Published:** 2023-05-12

**Authors:** Ghazwa B. Korayem, Lama Ali Alqahtani, Sultanah Hisham Alsulaiman, Abdullah M. Alhammad, Hisham A. Badreldin, Nora Alkhudair, Khalid Al Sulaiman, Ohoud Aljuhani

**Affiliations:** ^1^Department of Pharmacy Practice, College of Pharmacy, Princess Nourah bint Abdulrahman University, Riyadh, Saudi Arabia; ^2^Epidemiology and Biostatistics Section, Health Sciences Research Center, Princess Nourah bint Abdulrahman University, Riyadh, Saudi Arabia; ^3^Clinical Pharmacy Department, College of Pharmacy, King Saud University, Riyadh, Saudi Arabia; ^4^Pharmacy Practice Department, College of Pharmacy, King Saud bin Abdulaziz University for Health Sciences, Riyadh, Saudi Arabia; ^5^Pharmaceutical Care Services, King Abdulaziz Medical City, Riyadh, Saudi Arabia; ^6^King Abdullah International Medical Research Center, Riyadh, Saudi Arabia; ^7^Saudi Critical Care Pharmacy Research (SCAPE) Platform, Riyadh, Saudi Arabia; ^8^Department of Pharmacy Practice, Faculty of Pharmacy, King Abdulaziz University, Jeddah, Saudi Arabia

**Keywords:** clinical pharmacy faculty, direct patient care, clinical services, clinical pharmacists, faculty responsibilities

## Abstract

**Background:**

The quadripartite mission of clinical track faculty members involves research, teaching, services, and direct patient care. However, the extent of faculty involvement in direct patient care remains a challenge. Thus, the study’s objective is to evaluate the effort spent on direct patient care by clinical faculty of pharmacy schools in Saudi Arabia (S.A.) and identify factors that hinder or facilitate providing direct patient care services.

**Methods:**

This multi-institutional, cross-sectional questionnaire study conducted between July 2021 and March 2022 involved several pharmacy schools’ clinical pharmacy faculty members in S.A. The primary outcome was the percentage of time/effort spent on patient care services and other academic responsibilities. The secondary outcomes were the factors affecting the effort spent on direct patient care and the barriers preventing the provision of clinical services.

**Results:**

A total of 44 faculty members took the survey. The percentage of effort spent on clinical education was highest at a median (IQR) of 37.5 (30, 50), followed by that spent on patient care [19 (10, 28.75)]. The percentages of effort spent on education and the length of academic experience were negatively associated with efforts spent on direct patient care. The most commonly reported barrier affecting fulfilling patient care duties was the lack of a clear practice policy (68%).

**Conclusion:**

Although most clinical pharmacy faculty members were involved in direct patient care, half of them devoted only 20% or less of their time to it. An effective effort allocation for clinical faculty duties will require the development of a clinical faculty workload model that sets realistic expectations about the time spent on clinical and non-clinical duties.

## Introduction

The tripartite mission of any academician involves research, teaching, and services. However, clinical-track faculty members are expected to engage in additional clinical responsibilities as part of their daily duties. In the United States (U.S.), clinical-track faculty refers to practitioner educators who typically devote 50–70% and 30–40% of their effort to clinical practice and teaching or experiential training, respectively (1). Both of these obligations should be fulfilled by any clinical faculty member; however, based on experience and training, clinical faculty members typically devote more time to patient care than any other academic responsibilities (1, 2). Role conflict between patient care services and teaching obligations is reported as one of the common demands facing pharmacy faculty (3). A prospective cross-sectional survey involving 344 clinical faculty in the U.S. has reported that their mean (SD) percent effort spent on direct patient care is 30.8% (22.9) (2).

Similar to the U.S., clinical faculty members in Saudi Arabia (S.A.) usually enter the academic field after completing their postgraduate year one (general pharmacy practice) or year two residency training (specialized pharmacy residency), depending on the qualifications for the clinical assistant professor position. Unlike in the U.S., where there are two tracks for faculty positions (tenure and non-tenure), there is only one faculty track position in S.A. that is equivalent to the tenure track faculty in the U.S. In this track, faculty members are expected to participate more in research and academic/community services to become eligible for promotion (4). Also, the current model does not differentiate between clinical and non-clinical faculties. In S.A., the promotion criteria for clinical and non-clinical track faculty members heavily rely on publications, in addition to teaching and academic or community services. However, providing clinical services is not always among the criteria for the promotion. This may cause the clinical faculty members’ focus to shift from providing clinical services and training to attending duties that are more essential for promotion, such as research, teaching, and services.

Currently, most pharmacy schools in S.A. offer the Doctor of Pharmacy degree (PharmD) (5). This professional degree involves an intensive didactic scientific component paired with multi-level practical experiences. At the national level, the program learning outcomes are unified between these programs to ensure all students receive similar experiences. These programs require sufficient non-clinical and clinical faculty to produce competent professionals that will join the workforce. Clinical-track faculty members who provide direct patient care may also serve as clinical preceptors for students’ introductory or advanced training. Therefore, the pharmacy schools in S.A. have adopted a hiring and promotion pathway for clinical faculty members with professional training and relevant credentials, including licenses, to deliver scientific and experiential competencies in their academic programs. However, one of the challenges in pharmacy education in the S.A. is that the number of proficient clinical faculty members remains insufficient to meet the demand for instructors in clinical courses or for facilitating clinical training (6). Moreover, not all institutions are affiliated with clinical sites providing direct patient care services. Thus, this study aims to estimate the effort spent by clinical faculty of pharmacy schools in the S.A. on direct patient care and identify factors that hinder or facilitate direct patient care services.

## Methods

This multi-institutional, cross-sectional study involved clinical faculty members from several pharmacy schools in S.A., and it was conducted between July 2021 and March 2022. We included respondents who were full-time clinical faculty with advanced clinical training. The advanced clinical training for the clinical faculty includes faculty holding a master’s degree in clinical pharmacy, a general pharmacy residency, or a specialized pharmacy residency with or without a Ph.D. Non-clinical faculty members without clinical practice education or training, adjunct clinical pharmacy faculty members, or those working at pharmacy colleges outside S.A. were excluded.

This cross-sectional study was conducted in accordance with the Declaration of Helsinki. It was approved by the Institutional Review Board (IRB) of Princess Nourah bint Abdulrahman University (PNU) with IRB registration number HAP-01-R-059. Participant consent was obtained through their approval to participate in the survey.

### Outcomes

The primary outcome was the percentage of time/effort spent on patient care services and other academic responsibilities. The effort weight was reported in percentage, so the total weight of efforts spent on each academic responsibility, namely, education, research and scholarship, academic services, professional services, public services, and patient care, adds up to 100%. The secondary outcomes were the factors affecting the percentage of efforts spent on direct patient care, satisfaction with fulfilling academic and clinical duties, and the commonly reported factors (facilitators and barriers) affecting providing direct patient care duties and other academic duties.

### Survey design and distribution

A modified version of the validated questionnaire devised by Nutescu et al. was utilized with permission in this study (2). The modified questionnaire was validated by piloting the questionnaire to a convenient sample of 10 clinical practice faculty members from various pharmacy schools in S.A. The final survey instrument contained 67 questions, including open-ended and dropdown list questions. Some questions were branched, and follow-up questions would appear depending on the respondents’ answers.

The questionnaire was designed and implemented using REDCap. The survey web link was emailed using the Saudi Commission of Health Specialties (SCFHS) and the Saudi Society of Clinical Pharmacy (SSCP) portals. The respondents were assured of the anonymity of their responses and were informed that no individual or institutional identifiers would appear in the published results.

### Questionnaire sections

The modified questionnaire contains five sections covering different domains. The first section collected the respondents’ demographic information. The second section focused on faculty responsibilities, namely, clinical education (including didactic teaching and clinical training); research and scholarship; contribution to academic, professional, or public services; committee involvement or administrative work, consulting, professional organizations, and community services; and direct patient care or clinical service. The third section evaluated the pharmacy schools’ clinical practice infrastructure, such as an affiliation with a practice site, the existence of a collaborative agreement/policy with the practice site, academic load calculation, and clinical faculty responsibilities. The fourth section focused on the characteristics of the direct patient care practice site. In the last section, a Likert scale (i.e., strongly agree, agree, neutral, disagree, and strongly disagree) was used to evaluate the perception of the clinical faculty members regarding the availability of time to fulfill their academic duties. It also evaluated the limitations of the faculty in balancing their patient care or clinical duties with their other academic commitments. In addition, factors that motivate faculty members to provide patient care services apart from fulfilling their academic duties were assessed.

The clinical faculty responsibilities indicated in the survey were *clinical education* (i.e., didactic and clinical teaching), *research and scholarship*, and *contribution to academic, professional, or public services* (e.g., committees, administrative work, consultation, professional organization services, and volunteer services); and *patient care* (i.e., clinical pharmacy service).

### Statistical analysis and ethical consideration

We extracted all relevant information using the questionnaire entered in REDCapv version 7.3.6 hosted by PNU. It is an electronic data capture web application used to collect survey and non-survey data. The results of the pilot survey were excluded from the analysis. Descriptive analysis was used to summarize the data describing the characteristics of the faculty members and their institutions. The categorical variables were presented as numbers and percentages, whereas the continuous variables were presented as a median and interquartile range (IQR). An additional analysis was conducted to evaluate the statistical difference between the faculty members’ demographic characteristics, as well as the characteristics of the pharmacy schools and practice sites (independent variables) based on time/effort spent on patient care (patient care <20% vs. ≥20%). The cutoff percentage was based on the median percentage of time/effort spent by the faculty members (19%).

A Chi-square or Fisher exact test was used for categorical variables, and a Student’s *t*-test and Mann–Whitney U-test were used for the normally and non-normally distributed continuous variables, respectively. The reliable indepedent variable with *P*-values <0.1 in the bivariable analysis [e.g., time spent on education, academic services, and the length of academic experience, and clinical experience (in years)] were added in the model. Linear regression coefficients (β) with standard error (SE) were computed to determine the magnitude of associations of the independent variables with the time spent on patient care (dependent variable). The significance level was set at a *p-value* < 0.05 and the results were reported with a 95% confidence interval (CI). Data analysis was performed using Stata v17.0 (StataCorp LLC, United States).

## Results

### Demographics of the respondents

Out of the 56 respondents, only 44 were included in this study. Seven were excluded since they did not meet the inclusion criteria, two were duplicate responders, and three did not complete the survey’s major sections. Thus, the response rate was around 78.5%. The majority of the included respondents were females (61%) with a median age of 34.5 years. Most of the respondents were clinical faculty members with an academic title of assistant professors and above (74%) and were working in the central region of S.A. (59%). Most of the clinical faculty members either had completed a specialized pharmacy practice residency training or had an additional fellowship (69%), as shown in Table 1. As regards participation in academic and patient care, all (100%) of the respondents participate in clinical education or training, whereas only 88% participate in patient care or clinical practice. However, only 82% of the respondents reported participating in all four duties; clinical education, research, services, and patient care, as presented in Table 1.

**Table 1:** Demographic information of clinical faculty who participated in the survey.

Participant characteristics	*N* = 44
Age in years, median (IQR)	34.5 (34.5,36.75)
**Sex, *n* (%)**	
Male	17 (39)
Female	27 (61)
**Academic rank** *, *n* (%)	
Teaching assistant/ lecturer	8 (19)
Assistant Professor	29 (67)
Associate Professor	5 (12)
Professor	1 (2)
**Region, *n* (%)** *	
Central	26 (59)
Eastern	4 (9)
Western	10 (22)
Northern	2 (4.5)
Southern	2 (4.5)
**Pharmacy classification, *n* (%)**	
Pharmacist	9 (20)
Pharmacist I	18 (41)
Consultant	15 (34)
Not classified	2 (5)
**Advanced clinical training or education, *n* (%)**	
General pharmacy practice (PGY-1)	6 (14)
Specialized pharmacy practice (PGY-2)	10 (23)
Master’s in clinical pharmacy	4 (9)
Ph.D. in clinical pharmacy	1 (2)
PGY-1 and Ph.D. in clinical pharmacy	1 (2)
PGY-1 and master’s in clinical pharmacy	1 (2)
PGY-2 and fellowship	20 (46)
PGY-2 and master’s in clinical pharmacy	1 (2)
**Academic years of experience, median (IQR)**	5 (3, 10)
**Clinical years of experience after obtaining the advanced training/education, median (IQR)**	3.5 (2, 5.25)
**Hold an administrative position at the college, *n* (%)**	16 (36)
Dean	1 (5)
Vice dean	2 (11)
Head of the department	3 (17)
Head of a unit	6 (35)
Head of a program	2 (11)
Others	2 (11)
**Hold an administrative position at the practice site, *n* (%)**	13 (30)
Residency program director	9 (69)
Pharmacy director	1 (7.6)
Other	3 (23)
**Participation in academic and direct patient care, *n* (%)**	
Clinical education	44 (100)
Research and scholars	39 (88)
Administrative/college services	37 (84)
Direct patient care	39 (88)
**Participation in academic and direct patient care, *n* (%)**	
Education, research, services, and patient care	36 (82)
Education, research, and services	1 (2)
Education, research, and patient care	2 (5)
Education and patient care	1 (2)
Education	4 (9)

* Percentage out of 43, one missing answer.

### Faculty responsibilities and effort allocation

**Figure 1:**
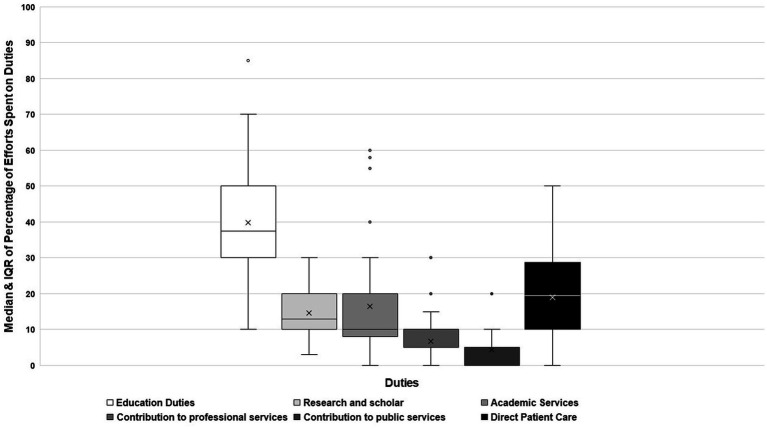
Percentage of effort spent on clinical and academic responsibilities among clinical faculty participated in the survey.

The highest percentage of time/effort was spent by faculty on clinical education, with a median (IQR) of 37.5 (30, 50), followed by that spent in clinical services [19 (10, 28.75)], in research and scholarship, [13 (10, 20)], and then in lasting contribution to public or professional services, as presented in Figure 1. As regards the proportion of time spent outside working hours to fulfill one’s academic and direct patient care, approximately half of the respondents (47%) reported spending 26–50% of their time doing their duties outside working hours, and 16% spent 51–75% of their time also outside working hours.

The reported median academic load per semester for assistant professors was 14 credit hours. Among the respondents, 75% provide student training, and 16% participate in residency training as part of their academic load, as shown in Table 2. Moreover, only 38% of the respondents reported that clinical services for patient care were included in their academic load.

**Table 2:** Faculty involvement in academic and clinical responsibilities and the reported structure of institutions.

Clinical education responsibilities	*N* = 44
**Academic load per semester, median (IQR)**	
Teaching assistant/lecturer	16.5 (45, 5–18)
Assistant Professor	14 (9.5, 16.5)
Associate Professor	14 (13, 17.5)
Professor	14 (14, 14)
**Students’ training included in the total academic load, *n* (%)**	
Yes	33 (75)
No	9 (20)
I do not know	2 (5)
Average of credit hours clinical training load, median (IQR)	5 (4, 6)
**Resident training included in the total academic load, *n* (%)**	
Yes	7 (16)
No	35 (79)
I do not know	2 (5)
Average of residency training load from the total academic load, median (IQR)	2 (2, 10)
Number of students trained per year, median (IQR)	18 (8.5, 30)
Number of residents trained per year, median (IQR)	1 (0, 4)
**Research and scholarship**	
Number of publications per year, median (IQR)	2 (1, 4.75)
Number of research participation per year, median (IQR)	2 (1, 2.75)
**Contribution to academic, public, and professional services**	
Number of committees serves, median (IQR)	2 (2–3.75)
**Clinical services responsibilities**	
*The academic load includes clinical services, n, (%)*	
Yes	17 (38)
No	25 (57)
I do not know	2 (5)
Average of credit hours clinical service load, median (IQR)	4.5 (1.8, 6)
Number of patients caring for per day, median (IQR)	9 (2, 14.75)
**University structure**	
Number of faculty members in the department, median (IQR)	30 (20, 42.5)
Number of clinical-track faculty members, median (IQR)	10 (7, 20)
Percentage of clinical track faculty, median (IQR)	38 (21, 66)
The presence of hospital-affiliated hospitals, n (%)	41 (93)
**The institution has a faculty pharmacy practice policy, *n* (%)**	
Yes	16 (36)
No	13 (30)
I do not know	15 (34)
P**rovides clear expectations of clinical services, *n* (%)**	
Yes	13 (29)
No	25 (57)
I do not know	6 (14)
**Provides faculty members dedicated time for clinical practice, *n* (%)**	
Yes	17 (39)
No	21 (47)
I do not know	6 (14)
I**ncludes clinical services in the academic load, *n* (%)**	15 (34)
Yes	15 (34)
No	24 (56)
I do not know	5 (11)
**Includes clinical services in the annual evaluation, *n* (%)**	3 (7)
Yes	3 (7)
No	30 (68)
I do not know	11 (25)
**Provides faculty member allowance for clinical services, *n* (%)**	5 (11)
Yes	5 (11)
No	33 (75)
I do not know	6 (14)
**Includes clinical services in the faculty promotion, *n* (%)**	
Yes	3 (7)
No	33 (75)
I do not know	8 (18)
**Practice site structure**	***N* = 38**
*The availability of clinical practice site, n (%)* *	
Yes	33 (86)
No	5 (13)
**Distance between the academic institution and clinical site, *n* (%)**	
Onsite <1 km	21 (55)
2–5 km	5 (13)
6–10 km	3 (8)
11–15 km	6 (16)
≥16 km	3 (8)
**Practice setting, *n* (%)**	
Acute care*	28 (74)
Primary care	5 (13)
Both inpatient and out-patient	5 (13)
**The clinical practice site fits the faculty member’s specialty, *n* (%)**	
Yes	36 (94)
No	2 (5)
**The availability of clinical pharmacist coverage during faculty other commitments, *n* (%)**	
Yes	24 (63)
No	13 (34)
I do not know	1 (3)
**Who to report to about faculty member’s clinical services, *n* (%)**	***N* = 33**
Hospital/clinic administrator	15 (45)
School/college of pharmacy administrator	2 (6)
Both	11 (33)
None	5 (15)
**Who is responsible for evaluating faculty member’s performance, *n* (%)**	***N* = 33**
Hospital/clinical administrator	9 (27)
Academic administrator	3 (9)
Both hospital/clinical administrators and an academic administrator	4 (12)
None	17 (52)

* Acute care settings include critical care, internal medicine, oncology, cardiology, infectious diseases, etc.

### Teaching and practice site characteristics

According to the respondents, one-third of the department personnel are clinical track faculty members with advanced clinical practice training or education. Most faculty members reported having hospital-affiliated practice sites (93%). However, 57% of the respondents mentioned there had been no clear expectations of the clinical services to be offered, 47% reported a lack of dedicated time for clinical practice, and 30% of the responding faculty reported the lack of a clear practice policy with the practice site (Table 2). As regards the location of the practice site, most of the respondents (68%) reported that their practice sites are in close proximity to their institution (<1–5 KM). Meanwhile, 52% of the respondents reported that no one was tasked to evaluate their clinical service performance, as shown in Table 2.

### Faculty fulfillment with academic and clinical responsibilities and factors affecting one’s ability to balance patient care with other academic duties

**Figure 2:**
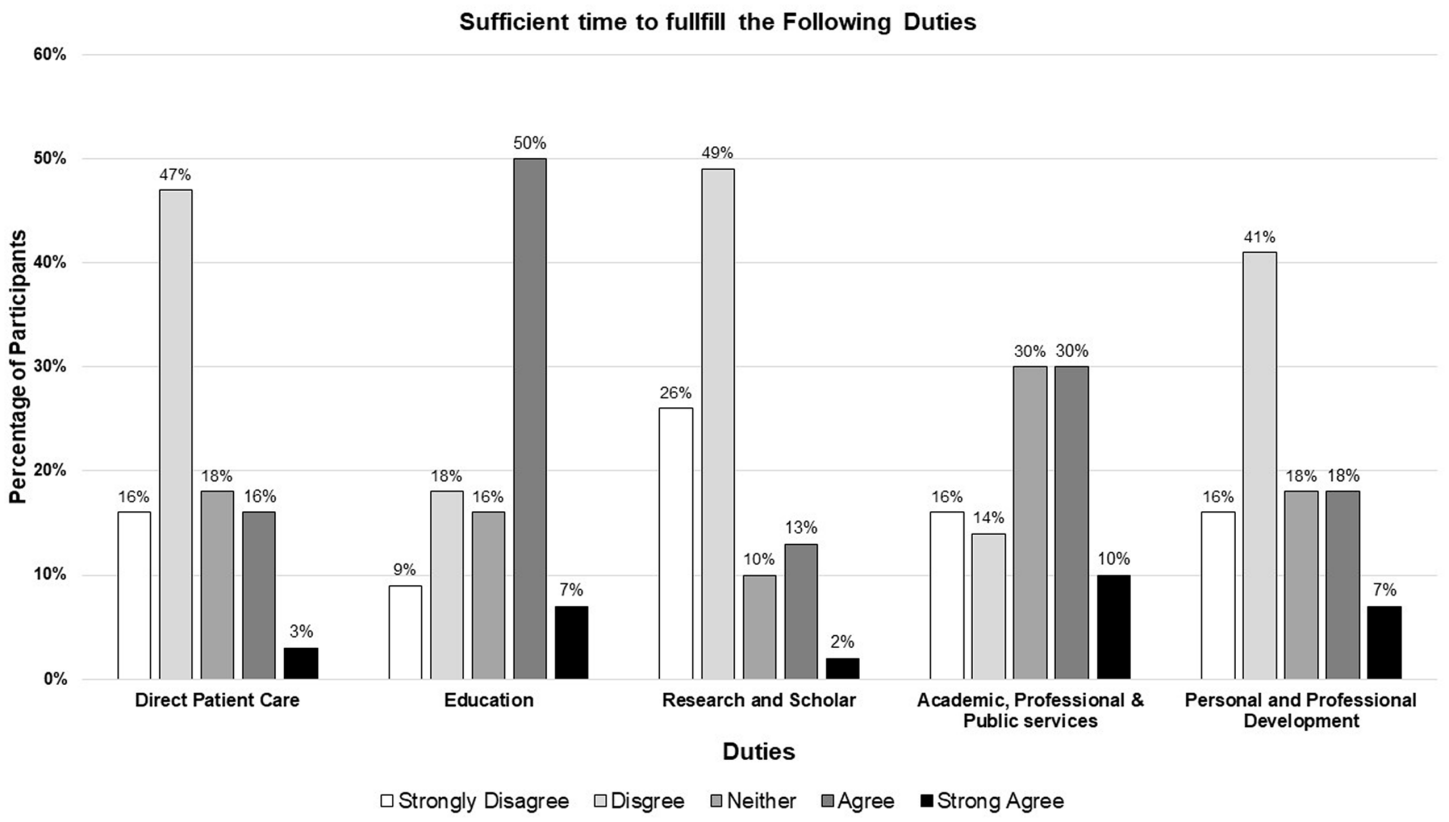
Clinical pharmacy faculty perceptions regarding the time allotted to fulfill each academic responsibility.

More than half of the respondents agreed or strongly agreed with having sufficient time to fulfill their educational duties (57%), whereas most of them strongly disagreed or disagreed with having sufficient time to participate in research and scholarship (75%) and direct patient care (63%), as depicted in Figure 2.

**Figure 3:**
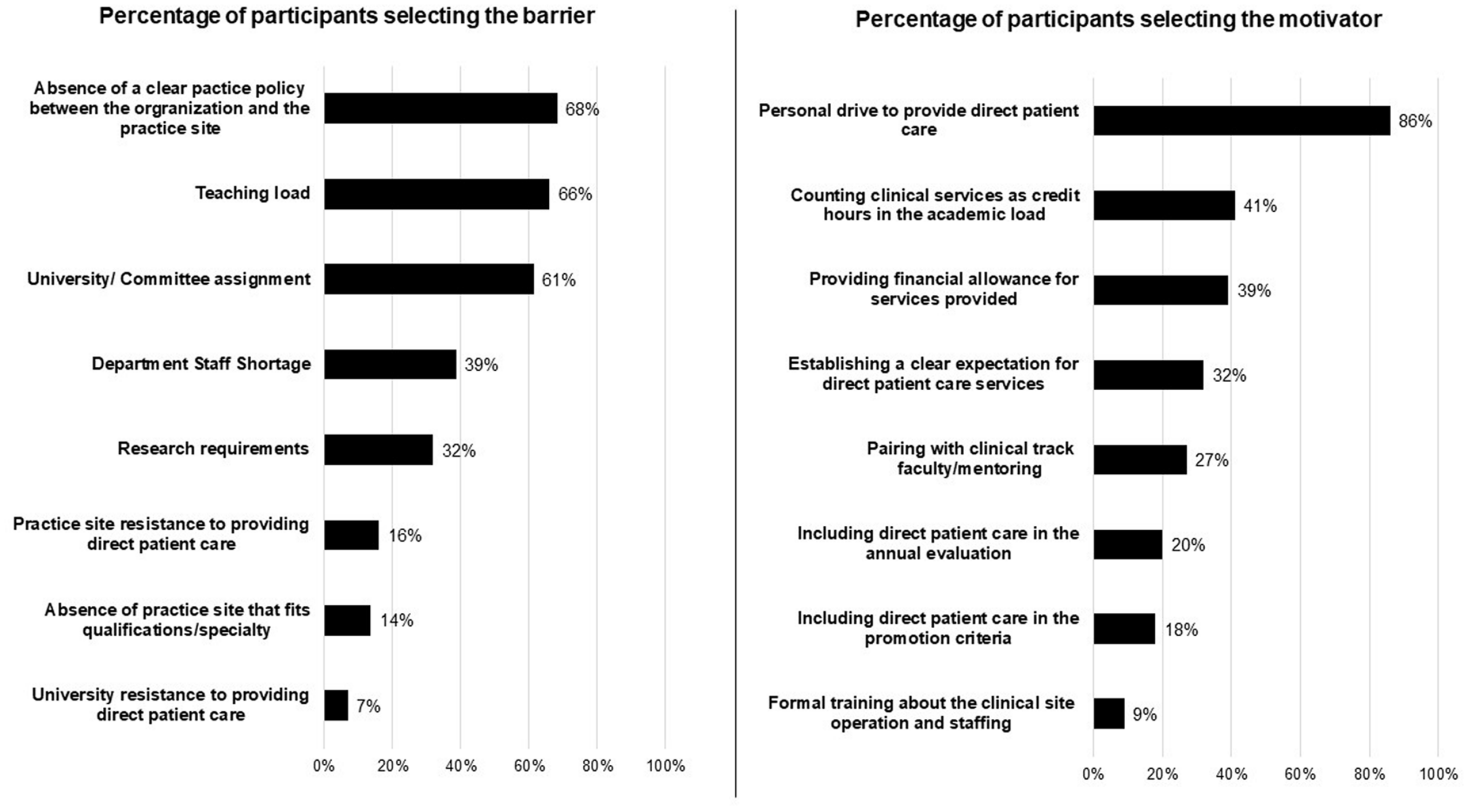
The number of Clinical pharmacy faculty perceived the following as barriers and motivators affecting providing direct patient care with other academic duties (number of responders *n* = 44).

The most commonly reported barriers that affect participating patient care duties, along with their other academic duties, were the lack of a clear practice policy between the academic institutions and the practice sites (68%), followed by teaching load (66%), and then university or committee assignments (61%), as presented in Figure 3. The other reported barriers were *“lack of evaluation on patient care services, lack of impact of providing patient care services on career promotion,” “limited number of full clinical pharmacists at the hospital,”* and *“lack of aligned schedule between the college and the hospital.”* By contrast, the most commonly reported motivators for providing patient care, along with other academic duties, were the belief that it is important for career development (90%), followed by personal drive to provide such services (86%), and then clinical services being counted as credit hours in the academic load (41%); motivators are presented in Figure 3. The other reported facilitators were *“gaining experience and building rapport with the medical team and other hospital staff members.”*

### Factors affecting the effort spent on patient care

The percentage of time/effort spent on education and the length of academic experience were significantly higher among the respondents who spent <20% of their time on patient care. By contrast, those who spent ≥20% of their time/effort on patient care had a institution expectations and had dedicated a greater amount of time to clinical services, as presented in the Supplementary Appendix 1. Moreover, this group had a significantly higher number of patient care load per day and number of poster presentations per year. The multivariable regression analysis showed that the higher percentage of time/effort spent on education, academic services, and the length of academic experience were negatively associated with the amount of time spent on patient care, as shown in Table 3. By contrast, a longer clinical experience (in years) was positively associated with the percentage of time spent on patient care.

**Table 3:** Factors affecting clinical-track faculty members spending <20% of their time/effort on patient care.

Factors	Coefficient (estimates) (95%CI)	*p*-value
Academic years of experience	−0.10 (−1.77, −0.26)	0.009
Clinical years of experience after obtaining the advanced training/education	0.94 (0.04,1.85)	0.04
Percentage of time/effort spent on education	−0.45 (−0.62,-0.28)	<0.01
Percentage of time/effort spent on academic services	−0.63 (−0.89, −0.37)	<0.01

## Discussion

Overall, the vast majority of respondents have fulfilled the minimum qualifications for clinical practice as defined by (7). Our survey showed that not all clinical track-faculty members are engaged in patient care. The median percentage of effort spent on direct patient care, among other academic duties, was around 20%. In contrast, a recent survey involving pharmacy practice faculty in the U.S. has shown that more than half of the clinical faculty members (63.8%) spend approximately 26–50% of their efforts on providing direct patient care (8).

In the current study, we found that longer academic experience and the percentage of effort spent on education and academic services were negatively associated with the time/effort spent on the direct patients care. This result was also supported by the respondents’ perception that teaching load and university assignments were the second and third most common barriers affecting balancing patient care with other academic duties. Moreover, studies have reported that academic appointments and didactic teaching workload prevent pharmacy practice faculty from engaging in direct patient care services (2, 8). Third of our respondents hold administrative positions at hospitals or academic institutions. These administrative appointments at academic institutions or practice settings could explain why clinical faculty members might spend less time on direct patient care as they gain more experience and advance in their careers.

As with any clinical pharmacist, clinical faculty members can significantly impact the community’s health outcomes. Previous studies have proven that clinical pharmacists’ involvement in patient care services in various settings improved patient outcomes, safety, and healthcare efficiency (9–11). However, the limited number of residency-trained clinical pharmacists involved in direct patient care in the academic settings in the S.A. negatively impacted the training and education of future pharmacists in the S.A. and, ultimately, patient outcomes (12). According to our study, the number of clinical pharmacists appointed by hospitals and the limited number of faculty members with advanced clinical training who work in academia may have an impact on how much time is spent providing direct patient care.

The lack of clear expectations in the provision of pharmacy care services is another challenge that could affect faculty members’ engagement in clinical practice (2, 3, 13). In the survey, respondents declared that no clear practice policy exists in their practice setting, which was also the most common barrier affecting the fulfillment of direct patient care duties. Uncertainty regarding the required extent of active engagement in direct patient care, the number of credits counted as academic load, and the method for evaluating patient care performance are some factors that must be considered to standardize the roles and responsibilities of clinical faculty members.

Our results showed that pharmacy faculty who are providing direct patient care services in S.A. strain to manage their clinical and non-clinical obligations. This challenge emphasizes the importance of creating clearly defined pharmacy clinical services and establishing achievable objectives in order to enhance faculty productivity and satisfaction. A multi-modal approach, for instance, was implemented by the College of Pharmacy at the University of Buffalo to assist clinical faculty members in achieving a balanced allocation of efforts (13). In that paradigm, clinical faculty members should devote roughly 30% of their time to teaching, 30% to clinical practice, 20% to research and scholarship, and 20% to service (13). Due to the significant differences in the infrastructure of clinical pharmacy practice and educational requirements in the S.A., this model may be difficult to implement.

The study also draws attention to the challenges clinical faculty members encounter in balancing their direct patient care responsibility with their other academic responsibilities. This problem was evident in our results since more than half of the respondents claimed insufficient time to handle direct patient care. Similarly, nearly 70% of the clinical faculty members in the U.S. admitted that they do not have enough time to fulfill their non-clinical and clinical responsibilities (2). Moreover, many respondents stated they had insufficient time to accomplish their research and scholarly obligations. Even though most of our study participants perceive that involvement in clinical duties could be a limitation for research enhancement, other faculty could perceive this as a great channel for research promotion that could be translated to improving health-related outcomes.

As shown in this study, the time spent on education negatively affected the time directed toward patient care. Therefore, it must be highlighted that clinical-faculty members’ provision of direct patient service is not a privilege they enjoy in their spare time or on demand (8). Clinical faculty members are accountable for their patients as much as they are accountable for their students. The involvement in patient care can give back to clinical education as the faculty’s clinical experience could enrich the scientific content delivered in clinical courses or laboratories. Also, there is a huge need for faculty to serve as preceptors for training students or residents. This role could help expand the availability of practice training seats when a limited number of training seats challenge most pharmacy schools in S.A. (14). Moreover, according to the Saudi Commission for Health Specialties (SCFHS), some faculty members who serve as residency program directors and/or as clinical preceptors must be full-time pharmacists at the practice site. Thus, setting realistic expectations about the time spent on direct patient care is essential to improve patient care and provide more effective didactic training and teaching strategies.

We propose several actions to help clinical faculty members fulfill patient care duties and devote more time to non-clinical academic activities. First, academic institutions must create a customized clinical faculty workload model that sets the minimum requirement for a balanced distribution of effort for clinical services, educational responsibilities, services, scholarly activities, and faculty development. Institutions can modify the model according to their specific needs (e.g., undergraduate education or postgraduate training) and faculty workload without falling below the minimum threshold for each responsibility. Integrating direct patient care within faculty workload expectations can help enhance faculty productivity and maintain research and scholarly activity, ultimately supporting their annual appraisal, promotion, and enhancing patient care through clinical research findings. Second, academic institutions must develop a collaborative practice agreement with their practice sites; this agreement governs the clinical practice of clinical faculty members and standardizes the definition of clinical pharmacy services.

To our best knowledge, this study is the first to evaluate the current status of direct patient care involvement of clinical pharmacy faculty members and balancing it among other academic duties. However, our study has several limitations. First, our sample size was relatively small. However, the available number of faculty members with advanced clinical training in some academic institutions in S.A. was quite low. Also, sample size calculation was difficult to predetermine because the total number of clinical faculty at all pharmacy schools in S.A. was not publicly unavailable. Thus, we tried to include a representative sample from different geographical regions, academic institutions, and academic ranks. In addition, there are a number of other biases, including reporting and recall bias, where many respondents might not be able to recall all the details required to answer the survey. Also, there was a potential for selection bias that may have occurred in which we have an overrepresentation of female faculty members. We conducted regression analyses to eliminate some of these biases to determine whether certain confounders could affect our results. This study could help develop a national and regional framework for clinical track faculty to actively engage in direct patient care services. However, future studies should re-evaluate our outcomes using a more broadly representative sample.

## Conclusion

Clinical pharmacy faculty members face challenges balancing direct patient care and academic responsibilities. The absence of a well-defined policy for clinical practice between educational institutions and practice sites often hinders direct patient care delivery. Incorporating patient care into faculty workloads can enhance productivity, promotion prospects, and overall quality of patient care. Collaborative efforts among faculty, institutions, and practice sites can create a more effective healthcare and education system.

## Data availability statement

The original contributions presented in the study are included in the article/Supplementary material, further inquiries can be directed to the corresponding author.

## Ethics statement

This study was approved by the Institutional Review Board (IRB) of Princess Nourah bint Abdulrahman University (PNU) with IRB registration number HAP-01-R-059. Written informed consent from the participants was not required to participate in this study in accordance with the national legislation and the institutional requirements.

## Author contributions

All authors listed have made a substantial, direct, and intellectual contribution to the work and approved it for publication.

## Funding

This work was supported by Princess Nourah bint Abdulrahman University Researchers Supporting Project number (PNURSP2023R78), Princess Nourah bint Abdulrahman University, Riyadh, Saudi Arabia.

## Conflict of interest

The authors declare that the research was conducted in the absence of any commercial or financial relationships that could be construed as a potential conflict of interest.

## Publisher’s note

All claims expressed in this article are solely those of the authors and do not necessarily represent those of their affiliated organizations, or those of the publisher, the editors and the reviewers. Any product that may be evaluated in this article, or claim that may be made by its manufacturer, is not guaranteed or endorsed by the publisher.

## Abbreviations

S.A.: Saudi Arabia
SCFHS: Saudi Commission for Health Specialties
U.S.: United States
SD: standard deviation
IQR: interquartile range
SE: standard error
PharmD: doctor of pharmacy degree
SSCP: Saudi Society of Clinical Pharmacy
PNU: Princess Nourah bint Abdulrahman University
