# Supplementary material for: Clinical Pharmacy Faculty Provision of Direct Patient Care, Challenges, and Opportunities
Source: Front Med (Lausanne). 2023 May 12;10:1143576. doi: 10.3389/fmed.2023.1143576 (PMC10213266; doi:10.3389/fmed.2023.1143576)
Supplement: Supplementary file 1 [file Table_1.DOCX]

Supplementary Material

**The Status of Patient Care Provision, Challenges, and Opportunities Among Clinical Track Pharmacy Faculty**

Ghazwa B. Korayem^1^,B.S.,PharmD

Lama Ali Alqahtani^1^,PharmD

Sultanah Hisham Alsulaiman, B.S, MSc^2^

Abdullah M. Alhammad, ,B.S.,PharmD^3^

Hisham A. Badreldin, ,B.S.PharmD, MPH^4,5,6^

Nora Alkhudair, ,B.S.,PharmD^3^

Khalid Alsulaiman,B.S,MBA^4,5,6,7^

Ohoud Aljuhani,PharmD ^8^

# Supplementary Table

**Appendix**

| **Table S1. Comparing clinical faculty who spent less than 20% of their time and efforts on patient care to who spent ≥ 20%** | | | |
| --- | --- | --- | --- |
| **Factors** | **Faculty spending <20% of time/effort on direct patient care (n =22)** | **Faculty spending ≥ 20% of time/effort on direct patient care (n =22)** | ***p* value** |
| **Age in years, mean (SD)** | 35 (34-36) | 34 (30-37) | 0.34 |
| **Sex, n (%)** |  |  |  |
| Male | 8 (36) | 9 (41) | 0.76 |
| Female | 14 (64) | 13 (59) |  |
| **Academic rank, n (%)** |  |  |  |
| **Teaching assistant/ lecturer** | 3 (14) | 5 (24) | 0.76 |
| **Assistant Professor** | 15 (68) | 14 (67) |  |
| **Associate Professor** | 4 (18) | 1 (5) |  |
| **Professor** | 0 (0) | 1 (5) |  |
| Region, n (%)* |  |  | 0.66 |
| Central | 13 (59) | 13 (59) |  |
| Eastern | 2 (9) | 2 (9) |  |
| Western | 4 (18) | 6 (27) |  |
| Northern | 2 (9) | 0 (0) |  |
| Southern |  |  |  |
| **Pharmacy Classification, n (%)** |  |  | 0.49 |
| Pharmacist | 5 (23) | 4 (18) |  |
| Pharmacist I | 8 (36) | 10 (45) |  |
| Consultant | 7 (32) | 8 (36) |  |
| Not classified | 2 (9) | 0 (0) |  |
| **Advanced clinical training or education, n (%)** |  |  |  |
| General pharmacy practice (PGY-1) | 6 (27) | 0 (0) | 0.010 |
| Specialized pharmacy practice (PGY-2) | 4 (18) | 6 (29) | 0.42 |
| Master's in clinical pharmacy | 2 (9) | 2 (9) | 0.96 |
| Ph.D. in Clinical Pharmacy | 0 (0) | 1 (5) | 0.15 |
| PGY-1 and Ph.D. in Clinical Pharmacy | 0 (0) | 1 (5) | 0.30 |
| PGY-1 and master's in clinical pharmacy | 0 (0) | 1 (5) | 0.30 |
| PGY-2 and fellowship | 9 (41) | 11 (52) | 0.45 |
| PGY-2 and master's in clinical pharmacy | 1 (5) | 0 (0) | 0.32 |
| Academic years of experience, mean (SD) | 9 (4-10) | 3 (2-6) | 0.026 |
| Clinical years of experience after obtaining the advanced training/education, mean (SD) | 4 (2-6) | 2.8 (2-5) | 0.43 |
| **Academic responsibilities involvement, n (%)** |  |  |  |
| Education | 22 (100) | 22 (100) |  |
| Research | 20 (91) | 19 (86) | 0.63 |
| College Services | 19 (86) | 18 (82) | 0.68 |
| Clinical Services | 19 (86) | 20 (91) | 0063 |
| **Percentage of time/ effort spent on, (%)** |  |  |  |
| Education | 47.5 (30-60) | 30 (25-40) | 0.047 |
| Research & Scholars | 15 (10-20) | 10.5 (10-20) | 0.98 |
| Administrative/ college services | 12.5 (10-25) | 10 (5-10) | 0.10 |
| Professional & public services | 5 (2.5-10) | 5 (3.5-7.5) | 0.70 |
| Patient care | 10 (5-10) | 27.5 (20-30) | <0.001 |
| **Involved in Administrative position** | 9 (41) | 7 (32) | 0.53 |
| **Number of patients caring for per day, mean (SD)** | 2 (1-14) | 10 (8-15) | 0.13 |
| **Number of research participation per year, mean (SD)** | 2 (1-4) | 2 (1-5) | 0.56 |
| **Number of posters per year, mean (SD)** | 1 (1-2) | 2 (1-3) | 0.028 |
| **Number of residents precepted per year, mean (SD)** | 0 (0-3) | 2.5 (0-4) | 0.074 |
| **Number of students trained precepted per year, mean (SD)** | 18 (6-30) | 18 (10-30) | 0.79 |
| **Number of the committee involved in per year, mean (SD)** | 2 (1-5) | 2 (2-3) | 0.86 |
| **University Factors** | | | |
| **Number of faculty members in the department, n (%)** | 30 (15-40) | 29 (20-50) | 0.45 |
| **Number of clinical-track faculty members, n (%)** | 10 (5-21.5) | 10 (10-20) | 0.67 |
| **The academic load includes clinical services, n, (%)** | 7 (32) | 10 (45) | 0.64 |
| **Students' training included in the total academic load, n(%)** | 17 (77) | 16 (73) | 0.93 |
| **Resident training included in total academic load , n(%)** | 3 (14) | 4 (18) | 0.92 |
| **The presence of hospital-affiliated hospitals, n (%)** | 20 (91) | 21 (95) | 0.55 |
| **The institution has a faculty pharmacy practice policy/collaborative agreement, n (%)** | 8 (36) | 8 (36) | 0.17 |
| **The institution provides clear expectations of clinical services, n (%)** | 3 (14) | 10 (45) | 0.033 |
| **The institution provides faculty members dedicated time for clinical practice, n(%)** | 8 (36) | 9 (41) | 0.027 |
| **The institution includes clinical services in the academic load, n (%)** | 7 (32) | 8 (36) | 0.057 |
| **The institution includes clinical services in the annual evaluation, n (%)** | 0 (0) | 3 (14) | 0.15 |
| **The institution provides faculty member allowance for clinical services, n (%)** | 0 (0) | 5 (23) | 0.051 |
| **The institution includes clinical services in the faculty promotion, n (%)** | 1 (5) | 2 (9) | 0.061 |
| **Practice site factors** | | | |
| The clinical practice site fits the faculty member's specialty, n (%) | 16 (72) | 20 (90) | 0.13 |
| **Distance between the academic institution and clinical site, n (%)** |  |  | 0.16 |
| Onsite < 1 km | 23 (67) | 9 (45) |  |
| 2-5 km | 3 (17) | 2 (10) |  |
| 6-10 km | 0 (0) | 3 (15) |  |
| 11-15 km | 1 (6) | 5 (25) |  |
| >/= 16 km | 2 (11) | 1 (5) |  |
| **The availability of clinical pharmacist coverage during faculty other commitments, n (%)** | 12 (67) | 12 (60) | 0.61 |
